# Simulation-Based Education in the Artificial Intelligence Era

**DOI:** 10.7759/cureus.40940

**Published:** 2023-06-25

**Authors:** Nobuyasu Komasawa, Masanao Yokohira

**Affiliations:** 1 Community Medicine Education Promotion Office, Faculty of Medicine, Kagawa University, Miki-cho, JPN; 2 Department of Medical Education, Faculty of Medicine, Kagawa University, Miki-cho, JPN

**Keywords:** artificial intelligence, technical skill, non-technical skill, medical education, simulation

## Abstract

Simulation-based medical education (SBME) has been widely implemented in skill training in various clinical specialties. SBME has contributed not only to patient and medical safety but also to undergraduate and specialist education in the healthcare field. In this review, we discuss the challenges and future directions of SBME in the artificial intelligence (AI) era. While SBME fidelity or methods may become highly complicated in the AI era, the fact is that learners play a central role. As SBME and clinical education are complementary, mutual feedback and improvement are essential, especially in non-technical skill development. For the development of sustainable SBME in the clinical field in the AI era, continuous improvement is needed by academia, educators, and learners.

## Introduction and background

Simulation-based medical education (SBME) has been widely utilized for acquiring technical or non-technical skills in various medical fields such as internal medicine, anesthesiology, emergency medicine, and surgery [1].

Until around 2000, when simulation education was not yet widespread in Japan, even clinical trainees were expected to perform professional procedures on patients. In addition, it was difficult to instruct the method in the presence of the patient during the procedure to avoid making the patient anxious. Simulation education is an epoch-making tool that can be used to solve these problems.

It has been about 60 years since systemic SBME, such as basic life support using manikins, was introduced. The classic simulator Laerdal Resusci Anne^TM^ was developed in the 1960s, which provided learners the opportunity to train in basic life support effectively. Another classic integrated simulator known as SimOne^TM^ was also developed. SimOne^TM^ contained various simulation abilities such as breathing, pulse detection, blood pressure measurement, and eye movements. This simulator also can respond to drugs and change the above reaction [2]. SBME has contributed to patient and medical safety, and specialist education in various clinical fields.

Over the past several decades, the broad adoption of SBME has been driven by patient or medical safety concerns. In other words, a large number of medical educators have struggled to improve the quality of patient safety or clinical outcomes by utilizing SBME [3]. Requirements for improved basic clinical skill training have contributed to the global development of a wide variety of clinical SBME programs. Simulations for clinical skill training have become popular with various positive educational effects all over the world, which is assuring the effectiveness or superiority of SBME [4].

SBME has contributed not only to patient or medical safety but also to undergraduate and specialist education in the healthcare field. Combined with the development of educational technology in medical education, SBME is now playing an essential role in clinical training for various medical professionals [5]. In recent years, artificial intelligence (AI) dramatically changed not only clinical practice but also education technology. Thus, SBME should adapt to these changes to facilitate clinical learning in the AI era.

In this review, we discuss the challenges and future directions of SBME in the AI era.

## Review

Simulation-based education for cultivating non-technical skills

Both technical and non-technical skills are essential for ensuring good clinical outcomes and patient or medical safety improvements. Non-technical skills are cognitive resource skills that contribute to efficient task performance [5]. Without non-technical skills, we cannot maximize the effectiveness of technical skills and provide skill domains such as situational awareness, decision-making, communication, and stress management.

Non-technical skills training is routinely incorporated as an element of patient or medical safety training programs including simulation. SBME is a valuable and effective instructional method for both technical skills and non-technical crisis management skills in various medical settings [6]. Experiences in simulated environments provide learners the opportunity to practice and acquire carious non-technical skills such as communication or rapid response skills [7].

Artificial Intelligence and Medical Systems

Third-generation AI based on deep learning has begun to provide various values in a variety of medical settings. AI is frequently applied to various complex tasks, which need the intellectual processes characteristic of humans such as reasoning, discovering, or learning from past experiences [8].

AI contributes to the curation and processing of big data storage into new meanings as well as the retention and analysis of medical information. Thus, information has begun to drive people to perform various tasks in a timely manner [9]. AI is expected to diminish the physicians’ burden in digital data interpretation and can improve their process for proceeding with diagnoses or prognoses.

In the near future, physicians may encounter patients in quite different healthcare contexts compared to the present [10]. Digitalized healthcare AI systems allow not only physicians but also patients to access clinical or basic medical information more easily. Thus, the role of the doctor may shift from diagnosis to patient mental care. There is a possibility that the humanistic aspect of medicine such as communication or the attitude toward care will come to be more emphasized because it is difficult to be performed by AI technology [11]. Like other specialties, a collaboration between physicians and AI technology contains the great potential to improve not only clinical decision-making but also patient health outcomes and happiness in the future medical environment.

Medical Education in the AI Era

While the application of AI comes to play a significant role in the medical and healthcare system, all educators should be aware of AI and its associated problems [12]. AI technology will likely dramatically change medical education from the viewpoint of accurate diagnosis or surgical procedure assistance. While AI can provide various positive effects in the present clinical environment, ethical or legal concerns arise inevitably. Medical schools all over the world are expected to develop AI and data science literacy as part of the educational curriculum. Furthermore, life-long learning competency development on AI and associated problems is warranted in present medical education curriculums [13].

While the expected outcome of medical students in controlling AI remains unclear at present, medical schools or associations worldwide have just begun to establish data science and AI curricula that can be applied to clinical situations.

There is one important about learning style in the AI era. AI technology is now changing learning methods through advanced technology dramatically. As AI can deeply change not only clinical medicine but also learning styles in medical education, it is also essential to assess the changes in medical students [14].

While AI facilitated big data analysis leading to differential diagnosis, there is a risk that AI technology may diminish critical thinking skills in physicians. For example, if AI technology performs clinical diagnosis and possible treatment, thinking competency in medical doctors is expected to weaken. Another problem is that AI can also make mistakes. If medical doctors cannot recognize the mistakes, a critical accident may occur. Thus, we should also develop a safety net system for accidents caused by AI.

**Figure 1:**
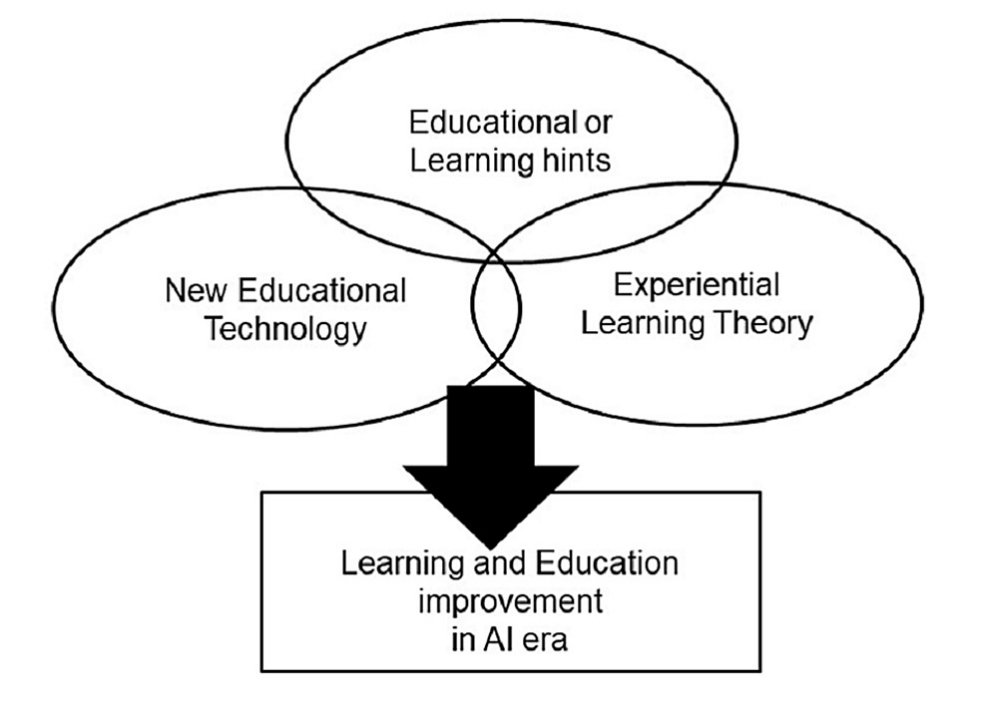
Learning and education improvement in the AI era This figure is the author's own creation.

Considering these limitations, we should implement a new medical education curriculum not only on the application or how to use it but also on a deep understanding of the principles and risks associated with AI. It is also important for medical students to acquire competency in solving associated future problems. In other words, novel non-technical skills for solving AI-induced problems are warranted in a data-driven medical environment. Integrative medical education committees should lead to continuous improvement and coordination to cultivate medical students to lead the healthcare system (Figure 1).

The Role of Simulation-Based Education in the AI Era

Various information and communication technology educational materials and remote classes were introduced, which were accelerated by the COVID-19 pandemic. Furthermore, severe inhibition of gathering-type simulation by the COVID-19 pandemic has also driven digital transformation in the SBME style [15]. For example, gathering-type resuscitation training, such as advanced life support utilizing a manikin or simulator, was suppressed, and virtual or augmented reality simulation was applied in some reports [15,16].

Entering the AI era, we are experiencing a dramatic change in both the clinical environment and medical education. SBME can be an effective method for cultivating sufficient non-technical skills in learners. However, we should also encounter some fundamental problems with SBME that should be solved, which can be accorded to the AI era. One possible problem is the redefinition of the simulator. As the function of the simulator increases and becomes more sophisticated, the cost also increases. Continuous update problems associated with AI programs are also anticipated in the case of virtual reality simulators. However, it is often difficult to show clear outcomes associated with medical safety or simulation-based education. Thus, stakeholders sometimes do not understand the necessity of simulators, even at present.

The most notable prerequisite for effective SBME include skill level consideration in learners, scenario or program development based on instructional design, and the facilitation skills of the instructor [2,4].

Even in the AI era, the most advanced simulator cannot have fidelity in all aspects, which consists of cognitive, psychological, environmental, and physiological ones [17]. Although an insufficiency of fidelity is often perceived as a limitation, it is a characteristic of SBME and is an advantage for educational outcomes accomplishment [18]. In summary, we should develop compliance in this field to overcome these inherent and complex problems in medical education in the AI era.

New Roles of SBME for Medical Schools

From the viewpoint of technical or non-technical skills, technical skill training may not change dramatically, even in the AI era, because the target skill is relatively simple and easy to imagine for both learners and educators. For example, clinical tracheal intubation or chest compression standard methods during resuscitation will not change dramatically, even in the AI era. Thus, technical skill acquisition in learners may not change, even though simulation fidelity develops innovatively. In contrast, the non-technical skill acquisition pathway may become complicated because AI-driven simulations can provide large amounts of information to learners at the same time. Thus, the non-technical skills expected to be obtained may become complicated, and instructors should be aware of the inherent risks associated with this.

Instructors of SBME should be familiar with both clinical practice and instructional skills [19]. Instructors who design and use SBME on specific themes should be prepared to evaluate the relationship to clinical skills, including the evaluation of learners’ technical and non-technical skills [20]. Simulation scenarios should incorporate elements of real clinical experiences, including some variations according to the situation or environment [21]. Simulation educators should also introduce learners to the purpose of simulation training, the basic knowledge needed to solve the problem, psychological safety, and the limitations of simulation fidelity, even in high-fidelity AI simulators.

Mutual Complement Between Simulation and Clinical Education

The SBME utility for acquiring technical or non-technical skills associated with fundamental clinical skills has been shown [22]. SBME training is effective in developing non-technical skills such as situational awareness, cooperation, decision-making, leadership, and communication in emergency situations [23]. The effectiveness of SBME is maximized when the simulation is closely aligned with clinical situations. Alignment of SBME to clinical situations can promote practical or concrete learning [24]. Likewise, effective debriefing and feedback have been identified as essential components of effective SBME, which is consistent with the adult learning theory. Even after AI simulation, sufficient debriefing and reflection provide learners with the opportunity to retain key technical or non-technical skills. In other words, the learners themselves, not AI, perform debriefing. We believe that experiential learning for competency-based training and assessment associated with mastery learning can be applied to medical education even in the AI era.

**Figure 2:**
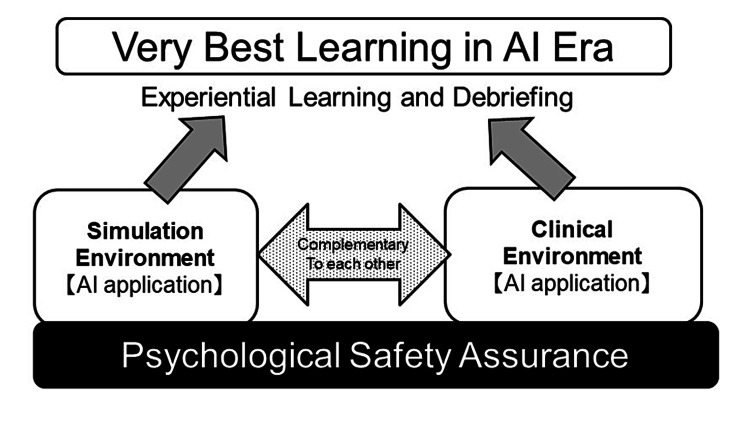
Complementary simulation-based and clinical education can produce the best learning in the AI era This figure is the author's own creation.

Both SBME and clinical education depend on the experiential learning theory, in which learners get simulated or real experience, then consider this deeply and obtain new lessons. From the viewpoint of the learners, they obtained real or simulated experiences. They produced new competencies after deep consideration (Figure 2).

While simulation-based education and clinical education depend on the experiential learning theory, the educational technology aspect of simulation-based education can provide sufficient feedback to clinical education.

Three basic points of simulation-based education, ‘basic knowledge acquirement’, ‘briefing,’ and ‘psychological safety assurance’ can be also applied to all clinical educators [25]. For example, the approval of competency by educators is very effective in developing self-fulfillment in learners. However, learners cannot overcome their barriers with only the approval of competency. To obtain sufficient competency, a lot of time and effort is required. Getting over their tasks or barriers is a competency of learners. Thus, educators should acknowledge or facilitate their efforts to develop self-esteem in learners.

Further Roles of Simulation-Based Education in the AI Era

New technologies are always accompanied by unknown risks associated with them. Some social or medical researchers suggest that medical learners should focus on active learning minds to cultivate research skills to control AI [26]. Some also suggest that in the future, diagnoses and treatment choices will be made mainly by AI, and human care is the most expected skill provided mainly by medical staff.

We believe that we should acknowledge that AI is not always correct and precise. For example, while AI-assisted robotic surgery provides the precision that is not impossible by human techniques, it also contains the inherent risk of unpredictable mistakes due to errors caused by the computed programs. Although it has been shown that AI applications can predict cardiac crises by electrocardiography [27], the prediction is not always guaranteed. Thus, it is essential for medical doctors to prepare not only to prevent such errors but also to respond rapidly to these errors. Future doctors must acquire competency regarding rescue method simulation in the case of accidental AI failure.

Because SBME and clinical education are complementary, mutual feedback and improvement are essential. For the development of SBME in the clinical medicine field, continuous improvement is needed by the academic society, educators, and learners.

## Conclusions

We reviewed the new role of SBME in the AI era. Although new technology has developed, learning theory has not changed rapidly. In the AI era, we should implement a new medical education curriculum not only on the application or how to use it but also on a deep understanding of the principles and risks associated with it. While new AI technology has developed, learning theory has not changed rapidly. Therefore, we should focus on the complementary role of SBME in clinical education and perform continuous improvements. For the development of sustainable SBME in the clinical field in the AI era, continuous improvement is needed by the academic society, educators, and learners. Therefore, we should focus on the complementary role of SBME in clinical education and perform continuous improvements.
